# The experiences of people with diabetes-related lower limb amputation at the Komfo Anokye Teaching Hospital (KATH) in Ghana

**DOI:** 10.1186/s13104-018-3176-1

**Published:** 2018-01-24

**Authors:** Vida Maame Kissiwaa Amoah, Reindolf Anokye, Enoch Acheampong, Helina Rubby Dadson, Mary Osei, Alberta Nadutey

**Affiliations:** 1 Centre for Disability and Rehabilitation Studies, Department of Community Health, Kwame Nkrumah University of Science and Technology, Kumasi, Ghana; 2 Department of Nursing, Garden City University College, Kumasi, Ghana

**Keywords:** Life experiences, Diabetes related amputation, Ghana

## Abstract

**Objective:**

Lower limb amputation not only causes major disfigurement, but renders people less mobile and at risk of loss of independence. Yet with appropriate rehabilitation, many people can learn to walk or function again and live high quality lives. This study sought to explore the experiences of patients with diabetes-related lower limb amputation at the Komfo Anokye Teaching Hospital. An exploratory study design was adopted using a qualitative approach and a purposive sampling to select 10 participants for the study. A semi-structured interview guide was used with an in-depth face-to-face interview. The interview was tape-recorded with an audio recorder while notes were taken in addition to the audio recording.

**Results:**

There were varying degrees of experiences ranging from physical as well as psychological and economic challenges. Amputees had to cope with playing entirely new roles after the amputation. They also experienced some economic challenges which were as a result of their inability to work. Some of the amputees consoled themselves with the fact that, despite their condition, they were better than other people. Others believed that whatever happened was Gods doing and nothing could be done about it. This self-consolation and the belief in God helped them to cope.

**Electronic supplementary material:**

The online version of this article (10.1186/s13104-018-3176-1) contains supplementary material, which is available to authorized users.

## Introduction

Amputation is the surgical removal of all or part of a limb or extremity such as an arm, leg, foot, hand, toe, or finger [1]. Lower limb amputation does not only disfigure, but renders people less mobile and may lead to loss of independence [2]. Despite advances in medicine and surgery, amputation continues to be a large problem among adults which has resulted in more than 900,000 people living with minor limb loss worldwide [3]. Most amputations occur as a result of Diabetes Miletus which is a metabolic disorder characterized by chronic hyperglycemia [4]. It is currently afflicting 194 million people worldwide [5]. The prevalence of diabetic foot ulcer ranges from 1.0 to 4.1% in the US, 4.6% in Kenya, 20.4% in the Netherlands [6, 7], between 11.7 and 19.1% in Nigeria [8, 9] and 20% in Iran [10]. Diabetic Foot Ulcer may become more common in the Tropics with the increasing prevalence of diabetes in Nigeria and Ghana [11]. It is believed that every 30 s a lower limb is lost somewhere in the world as a consequence of diabetes [12]. Diabetic foot complications are a leading cause of amputation and mortality in diabetic patients [13].

Lower limb amputation at the Komfo Anokye Teaching Hospital comprised of knee amputation (n = 23; 22.1%), Rays amputation of the toe (n = 15; 14.4%) and Hip de-articulation (n = 2; 1.9%) [14]. It is estimated that about 145,299 people have been amputated in Ghana [15].

Amputations are devastating because of the sudden change in body form and the difficulties associated with rehabilitation especially in developing countries like Ghana where prosthetic services are scarce [16]. Little has been done on the experiences of patients who have undergone diabetes-related lower limb amputation. This study was conducted to explore the experiences of persons living with diabetes related amputations to serve as a blueprint for further conclusive studies.

## Main text

### Methods

The study was conducted at Komfo Anokye Teaching Hospital (KATH) in Kumasi, Ashanti region, Ghana. It is the second largest hospital in Ghana and the only tertiary health institution in the Ashanti region. The hospital has a child health, diagnostic, pharmacy, physiotherapy, oncology, polyclinics and diabetes units.

An exploratory study was conducted to serve as a springboard upon which further studies could be conducted and a qualitative research approach was used. Patients were selected if they had been amputated at the foot (including toes or partial foot); at the ankle (ankle disarticulation); below the knee (transtibial); at the knee (knee disarticulation); above the knee (trans femoral) and at the hip (hip disarticulation). Data were collected between December 2016 and March 2017.

Patients were selected if they were between the ages of 25–70 years (most patients or cases for non-traumatic amputation fell between this age range); spoke English, Fante and Twi (the languages the investigators could speak) and were present at the hospital at the time of data collection. Patients were excluded if they could not speak Twi, Fante or English; could not give their consent to be part of the study; had traumatic amputation and were under 25 years and above 70 years.

A Purposive sampling technique was used to recruit participants. Participant recruitment continued until saturation was achieved with the 10th participant where no new significant information was obtained. The variability, experiences and perceptions narrowed by the time the 10^th^ participant was interviewed. Also, the study addressed an obvious, clear topic, and the information was easily obtained from the participants, therefore fewer participants were needed.

A semi-structured interview guide which was designed for the purpose of this study was used for the study. The instrument was developed based on the research questions and 8 questions were asked. The interview was tape-recorded with an audio recorder in addition to field notes. All interviews were conducted face-to-face at the orthopedic unit of the Komfo Anokye Teaching Hospital and in the participant’s homes, at a date and time which was convenient for them. The interviews were conducted in English, Fante and Twi and conducted by two of the investigators who have been trained on qualitative data collection. The interviews were pretested at Kumasi South Hospital using 5 participants. The pretest was used to evaluate the language competency and content validity of data collection materials and to estimate time length of full interview delivery as well as mark periods of respondent participant’s fatigue.

The tape-recorded interviews were transcribed verbatim and later categorized into similar themes. The analyses was done manually using the thematic content analysis guided by Miles and Huberman’s framework for thematic content analysis.

The authors divided the major themes into two categories which were physical experiences and coping strategies previously determined by the nature of the interview guide (Additional file 1).

## Results

### Demographic characteristics of participants

More males were part of the study as compared to females. Out of the 10 participants, 60% were aged 41–60 years; 70% were married and had attained formal education as shown in Additional file 2: Table S1.

### Physical experiences

#### Adjustment to amputation and present physical state

Adjustment to amputation was difficult for a lot of the amputees as most of them found it difficult to accept their present physical state due to low adjustment rate and the difficulty in using walking aids. Others perceived their condition as better compared to previous condition.

*“The feeling was bad; I always depend on other people to do everything”.* (Male Amputee 1).

*“I felt good after my legs were amputated compared to when the wound was there”.* (Male Amputee 3).

*“When I wake up and do not find anybody at home, I wait till they are back before doing anything”.* (Female Amputee 4).

#### Changes in lifestyle

Amputation changed patient’s lifestyle and the males were particularly worried because they had to abandon their jobs because they were physically demanding which their current condition could not meet.

*“…As you can see, am the only person left in the house, I sleep and wake and use clutches to go out because I was asked to exercise…”* (Male Amputee 4).

#### Loss of everyday functional independence

Functional independence makes it possible to perform daily living tasks without help. Participants who had been amputated did not have the ability to perform daily living tasks without the help of others. The female amputees were not happy with such limitations.

*“If you want to bath unless someone fetch the water for you. Since I came I haven’t gone to the bath or fetch water on my own….”* (Female Amputee 2).

#### Changes in family responsibilities/duties

Participants stated that the roles that they played in the family has changed because they could no longer play work and paying utility bills.

*“I have to go to work but I can’t and now am unemployed, my family’s feeding and education of my children are in the care of the woman and that is a problem…”* (Male Amputee 5).

#### Psychological/emotional experiences

Amputation had some psycho-emotional impact such as feeling of being a burden, unimportant and useless on patients and their families.

*“My wife knew that some people with diabetes had been amputated and didn’t live for long, so when it happened she asked the doctor if I can survive”* (Male Amputee 6).

*“It is difficult because I have become a burden because people look after me which makes me somehow useless….”* (Female Amputee 4).

#### Economic experiences

The survival of patients through the amputation process and afterwards is strongly dependent on the availability of financial resources. Patients experienced economic challenges which were as a result of their inability to work. One echoed that;

*“It affected me because I lost my job and can no longer cater for my family which is a big problem for me…*” (Male Amputee 2).

### Coping strategies

#### Self-consolation

Some of the participants consoled themselves with the fact that, despite their condition, they were better than other people. This self-consolation helped them to cope.

*“I take consolation in the fact that someone cannot even see. I say to myself look someone cannot see but if I can it should serve as encouragement for me…”*(Female Amputee 1).

#### Trusting in God

Some of the amputees trusted in God, they believed that whatever happened was Gods doing and nothing could be done about it.

*“I have faith in God, He created me and knows what is good for me so I have accepted it…”* (Female Amputee 3).

#### Dependence on immediate family members

The immediate Family members, spouse and children provided support in the form of advice, financial assistance and helped amputees in managing the home.

*“My family and children were very supportive financially and they are still taking care of me…”* (Male Amputee 4).

## Discussion

The study explored the experiences of individuals with diabetes-related lower limb amputation at the Komfo Anokye Teaching Hospital (KATH). The findings revealed that most of the amputees were males which corresponds with several findings in West Africa [17–21]. It was evident that some of the amputees struggled with walking aids because it was something entirely new. This is similar to another study which revealed that basic movement and activities was a challenge for most victims of amputation [22]. However, in a study by Godlwana [23], people with Lower Limb Amputation’s in the Johannesburg metropolitan area had no problem with mobility.

Amputation of a limb does not leave just a physical dent on an individual, but loads of psychological and emotional effects thus feeling useless because of the inability to do anything. This corroborates with the findings of De Godoy et al. [24] who found that the quality of life was generally lower for amputees. Surprisingly, some few amputees were not emotionally depressed as they were even happy for the loss of limb. According to Rybarczyk et al. [25], after many years of suffering with chronic debilitating diabetic foot ulcers, an amputation may result in improved quality of life for the patient, therefore individuals who undergo a lower limb amputation may not view it as a negative event.

Amputations had socio-economic effects due to loss of jobs and financial demands for medical care after surgery. Burger and Marincek [26] indicated that people with a lower limb amputation have problems returning to work.

This study found that not all amputees coped well with the condition as expected and this was supported by a study that lack of proper coping strategies could lead to psychological breakdown and affect management of the condition [27, 28]. They believed that their situation was willed by God and expressed their gratitude to their creator for granting them life. A positive approach to conditions of amputees has a positive correlation with pain and total recovery [29–31]. Amputees relied on family and friends for their day-to-day activities and survival. The change in lifestyle due to physical incompleteness which may lead to anxiety and depression calls for the need for adequate support and rehabilitation.

## Conclusion

Amputees had some physical experiences after the amputation. The males were worried as they could not work and cater for their families. Coping strategies were mostly, acceptance of current physical condition whiles others consoled themselves by comparing themselves to other people whom they perceived to be better than and some believed that whatever happened was Gods doing. Amputees were mostly dependent on spouse and children who provided support in various ways. This study will therefore contribute to the studies related to this area and provide a solid springboard for future studies (Additional file 3).

## Recommendations

The study recommends proper and adequate counseling of prospective amputees by the hospital staff such that they will be well prepared for the changes that are likely to occur after amputation.

Also, Amputees should come together and form self-help groups to develop coping strategies and share problems together.

## Limitation

The study design does not allow for a conclusive outcome and data was collected from a small sample which may have limited the chances of having varied responses. Whilst there may be limitations inherent in the study design and methods used, these by no means compromise the results reported. Being a qualitative study with the small sample size and mostly based on subjective answers limits the extent to which these findings can be generalized.

## Additional files

**Additional file 1.** Instrument used for data collection.

**Additional file 2: Table S1.** Demographic characteristic of participants.

**Additional file 3.** Change of authorship form.

## Abbreviation

KATH: Komfo Anokye Teaching Hospital
