# Supplementary material for: The experiences of people with diabetes-related lower limb amputation at the Komfo Anokye Teaching Hospital (KATH) in Ghana
Source: BMC Res Notes. 2018 Jan 24;11:66. doi: 10.1186/s13104-018-3176-1 (PMC5781296; doi:10.1186/s13104-018-3176-1)
Supplement: Supplementary file 1 — Additional file 1. Instrument used for data collection. [file 13104_2018_3176_MOESM1_ESM.docx]

**INTERVIEW GUIDE**

**SECTION A**

**Demographic profile**

Age ……

Gender Male [ ] Female [ ]

Marital status Single [ ] Married [ ] Divorced [ ] Widow/Widower [ ]

Religion Christian [ ] Muslim [ ] Traditionalist [ ]

Educational status Formal [ ] Informal [ ]

Occupation........................................................

Number of years diagnosed with diabetes......................

**SECTION B**

**OPENING QUESTION**

Please can you share with me your story after you have gone through amputation?

………………………………………………………………………………………..

Please could you please describe how you came to lose a limb?

……………………... …………………………………………………………………

**Sub questions**

1. Please tell me some of the physical experiences you have gone through after the amputation

Probe:

- How do you feel after the amputation?
- Physical appearance
- Body image
- Changes in lifestyle
- Complications/ side effects

1. Please share with me some of the emotional changes or responses after the amputation

Probe:

- How did you react before and after the amputation?
- How did your significant take you?
- How did it affect your personality?

1. Did you receive any support from your partner?

Probe:

- Family support
- Friends
- Community
- Spiritual counsellor
- Hospital physiotherapist
- Workplace
- Others

1. In what ways have the amputation changed or affected your life

Probe

- Work/ occupation/ role
- Family
- Marriage/partner/sexual relationship
- Financial issues
- Friends
- Others

1. Please can you tell me how you are coping after the limb loss?

- Physical coping strategies
- Psychological coping strategies
- Spiritual coping strategies

1. Please share your thoughts and feelings with me about what it has been like for you after the amputation
2. How do you see yourself now after this amputation?
3. Do you have any other experience you would want to share?

Thank you for your cooperation
