# Supplementary material for: The experiences of people with diabetes-related lower limb amputation at the Komfo Anokye Teaching Hospital (KATH) in Ghana
Source: BMC Res Notes. 2018 Jan 24;11:66. doi: 10.1186/s13104-018-3176-1 (PMC5781296; doi:10.1186/s13104-018-3176-1)
Supplement: Supplementary file 2 — Additional file 2: Table S1. Demographic characteristic of participants. [file 13104_2018_3176_MOESM2_ESM.docx]

**TABLE S1 Demographic characteristic of participants**

| ***Variable*** | ***Category*** | ***Frequency*** | ***Percentage*** |
| --- | --- | --- | --- |
| Sex | Male | 6 | 60 % |
|  | Female | 4 | 40 % |
| Age | 20-40years | 1 | 10 % |
|  | 41-60years | 6 | 60 % |
|  | 61-70years | 3 | 30 % |
| Marital status | Married | 7 | 70 % |
|  | Divorced | 2 | 20 % |
|  | Widow/ widower | 1 | 10 % |
| Educational Level | No formal education | 3 | 30 % |
|  | Formal education | 7 | 70 % |
| Duration for diabetes diagnosis | Less than a year | 0 | 0 |
|  | 1-5years | 0 | 0 |
|  | 6-10years | 4 | 40 % |
|  | More than 10years | 6 | 60 % |
| Religion | Christian | 7 | 70 % |
|  | Muslim | 3 | 30 % |
