# Supplementary material for: The experiences of people with diabetes-related lower limb amputation at the Komfo Anokye Teaching Hospital (KATH) in Ghana
Source: BMC Res Notes. 2018 Jan 24;11:66. doi: 10.1186/s13104-018-3176-1 (PMC5781296; doi:10.1186/s13104-018-3176-1)
Supplement: Supplementary file 3 — Additional file 3. Change of authorship form. [file 13104_2018_3176_MOESM3_ESM.pdf]

BioMed Central
The Open Access Publisher

Change of authorship request form

Please read the important information on page 4 before you begin

This form should be used by authors to request any change in authorship. Please fully complete all sections. Use black ink and block capitals and provide each author's full name with the given name first followed by the family name.

Section 1 Please provide the current title of manuscript

Manuscript ID no. RESN-D-17-01183R2

TITLE: The experiences of people with diabetes-related lower limb amputation at the Komfo Anokye Teaching Hospital (KATH) in Ghana

Section 2 Please provide the current authorship, in the order shown on your manuscript.

	First name(s)	Family name
1st author	Vida	Maame Kissiwaa Amoah
2nd author	Reindolf	Anokye
3rd author	Enoch	Acheampong
4th author	Helina	Rubby Dadson
5th author	Mary	Osei
6th author		
7th author		

Please use an additional sheet if there are more than 7 authors.

Section 3: Please provide a justification for change. Please use this section to explain your reasons for changing the authorship of your manuscript. Please refer to the journal policy pages for more information about authorship. Please explain why omitted authors were not originally included on the submitted manuscript.

There has not been any change in authorship, however, there was an oversight during the filling of the submission system where the details of the 6th author were not captured but her name was part of the list of authors on the manuscript. We only noticed it through the email we received from the editor. We strongly believe it was an oversight during the submission process.

The sixth author has been a part since day one and it is quite unfortunate her details could not be entered in the system during the submission. Maybe I thought all the names and details of the authors had been entered in the system only for me to realise that it was not the case through your email that you sent. I have now updated the author list in the system.

Scanned by CamScanner

BioMed Central
The Open Access Publisher

Change of authorship request form

Section 4 Proposed new authorship. Please provide your new authorship list in the order you would like it to appear on the manuscript

	First name(s)	Family name (this name will appear in full on the final publication and will be searchable on PubMed and similar databases)
1st author	Vida	Maame Kissiwaa Amoah
2nd author	Reindolf	Anokye
3rd author	Enoch	Acheampong
4th author	Helina	Rubby Dadson
5th author	Mary	Osei
6th author	Alberta	Nadutey
7th author		

Please use an additional sheet if there are more than 7 authors.

Section 5 Author contribution, Acknowledgement and competing interests section. Please use this section to provide revised Author Contribution, Acknowledgement and/or Competing Interests sections of your manuscript, ensuring you state what contribution any new authors made and, if appropriate acknowledge any contributors who have been removed as authors. Please ensure these are updated in your manuscript.

New Competing Interests statement: Not Applicable

New Author Contributions statement: Not applicable

New Acknowledgement Section: Not applicable

State 'Not applicable' if there are no new authors.

Scanned by CamScanner

BioMed Central
The Open Access Publisher

Change of authorship request form

Section 6 Declaration of agreement. *All* authors, unchanged, new and removed *must* sign this declaration.

* please delete as appropriate. Delete all of the bold if you were on the original authorship list and are remaining as an author

	First name	Family name		Signature	Affiliated institute	Date
1st author	Vida	Maame Kisiwaa Amoah	I agree to the proposed new authorship shown in section 4	[illegible]	Garden City University College, Kumasi, Ghana	8/01/2018
2nd author	Reindolf	Anokye	I agree to the proposed new authorship shown in section 4	[illegible]	Kwame Nkrumah University of Science and Technology, Kumasi, Ghana.	8/01/2018
3rd author	Enoch	Acheampong	I agree to the proposed new authorship shown in section 4	[illegible]	Kwame Nkrumah University of Science and Technology, Kumasi, Ghana.	7/01/2018
4th authors	Helina	Rubby Dadson	I agree to the proposed new authorship shown in section 4	[illegible]	Garden City University College, Kumasi, Ghana	8/01/2018
5th author	Mary	Osei	I agree to the proposed new authorship shown in section 4	[illegible]	Garden City University College, Kumasi, Ghana	8/01/2018
6th author	Alberta	Nadutey	I agree to the proposed new authorship shown in section 4 and the addition of my name to the authorship list	[illegible]	Kwame Nkrumah University of Science and Technology, Kumasi, Ghana.	8/01/2018
7th author			I agree to the proposed new authorship shown in section 4 **/and the addition/removal*of my name to the authorship list.**			

Please use an additional sheet if there are more than 7 authors. * please delete as appropriate. Delete all of the bold if you were on the original authorship list and are remaining.

Scanned by CamScanner

BioMed Central
The Open Access Publisher

Change of authorship request form

Important information. Please read.

- Please return this form, fully completed, to the editorial office. We will consider the information you have provided to decide whether to approve the proposed change in authorship. We may choose to contact your institution for more information or undertake a further investigation, if appropriate, before making a final decision.

- Please note, we cannot investigate or mediate any authorship disputes. If you are unable to obtained agreement from all authors (included those who you wish to be removed) you must refer the matter to your institution(s) for investigation. Please inform us if you need to do this.

- If you are not able to return a fully completed form within **14 days** of the date that it was sent to the author requesting the change, we may have to reject your manuscript. We cannot publish manuscripts where authorship has not been agreed by all authors (including those who have been removed).

- Incomplete forms will be rejected.

Scanned by CamScanner
